# Different peripheral expression patterns of the nicotinic acetylcholine receptor in dementia with Lewy bodies and Alzheimer’s disease

**DOI:** 10.1186/s12979-023-00329-9

**Published:** 2023-01-17

**Authors:** E. Costantini, C. Carrarini, P. Borrelli, M. De Rosa, D. Calisi, S. Consoli, D. D’Ardes, F. Cipollone, M. Di Nicola, M. Onofrj, M. Reale, L. Bonanni

**Affiliations:** 1 Department of Medicine and Aging Sciences, University “G. d’Annunzio”, Via dei Vestini, 66100 Chieti, Italy; 2 Department of Neuroscience, Imaging, and Clinical Sciences, “G. d’Annunzio” University of Chieti-Pescara, Chieti, Italy; 3 Department of Medical, Oral and Biotechnological Sciences, Laboratory of Biostatistics, University “G. d’Annunzio”, Via dei Vestini, 66100 Chieti, Italy; 4 Department of Innovative Technologies in Medicine and Dentistry, University “G. d’Annunzio”, Via dei Vestini, 66100 Chieti, Italy

**Keywords:** Dementia with Lewy bodies, Alzheimer’s disease, Cholinergic impairment, Nicotinic receptors, Acetylcholine esterase

## Abstract

**Background:**

The diffuse distribution of nicotinic cholinergic receptors (nAChRs) in both brain and peripheral immune cells points out their involvement in several pathological conditions. Indeed, the deregulated function of the nAChR was previously correlated with cognitive decline and neuropsychiatric symptoms in Alzheimer’s disease (AD) and Dementia with Lewy bodies (DLB).

The evaluation in peripheral immune cells of nAChR subtypes, which could reflect their expression in brain regions, is a prominent investigation area.

**Objectives:**

This study aims to evaluate the expression levels of both the nAChR subunits and the main known inflammatory cytokines in peripheral blood mononuclear cells (PBMCs) of patients with DLB and AD to better characterize their involvement in these two diseases.

**Results:**

Higher gene expression levels of TNFα, IL6 and IL1β were observed in DLB and AD patients in comparison with healthy controls (HC). In our cohort, a reduction of nAChRα4, nAChRβ2 and nAChRβ4 was detected in both DLB and AD with respect to HC. Considering nAChR gene expressions in DLB and AD, significant differences were observed for nAChRα3, nAChRα4, nAChRβ2 and nAChRβ4 between the two groups. Moreover, the acetylcholine esterase (AChE) gene expression was significantly higher in DLB than in AD. Correlation analysis points out the relation between different nAChR subtype expressions in DLB (nAChRβ2 vs nAChRα3; nAChRα4 vs nAChRα3) and AD (nAChRα4 vs nAChRα3; nAChRα4 vs nAChRβ4; nAChRα7 vs nAChRα3; nAChRα7 vs nAChRα4).

**Conclusions:**

Different gene expressions of both pro-inflammatory cytokines and nAChR subtypes may represent a peripheral link between inflammation and neurodegeneration. Inflammatory cytokines and different nAChRs should be valid and accurate peripheral markers for the clinical diagnosis of DLB and AD. However, although nAChRs show a great biological role in the regulation of inflammation, no significant correlation was detected between nAChR subtypes and the examined cytokines in our cohort of patients.

## Introduction

The Central Nervous System (CNS) has been considered for many years as an immune-privileged area protected by the blood-brain barrier (BBB), but this concept has been dismissed by the identification of extensive interaction between the peripheral and brain immune systems [1]. Indeed, the cytokines produced by the peripheral immune cells can cross the BBB determining microglia and astrocyte activation [2], whereas cytokines produced by CNS cells, including astrocytes and neurons, can cause neuroinflammation after the stimulation of peripheral cytokines [3, 4].

Neuroinflammation represents an initial beneficial mechanism that defends the CNS from different pathogenic agents. However, it can subsequently contribute to a neurodegenerative process for its continuous inflammatory response to endogenous and exogenous factors [5].

Inflammation, as well as neurodegeneration, may be related to cholinergic dysfunction, which is also implicated in neuron-glia interactions. The cholinergic system plays its function by the neurotransmission of acetylcholine (ACh), which is facilitated by the binding of both muscarinic and nicotinic receptors (nAChR) [6].

The involvement of the cholinergic system in these two processes, such as inflammation and neurodegeneration, is mainly related to the presence of the nAChRα7. Indeed, the binding of ACh, released from the basal forebrain nuclei to nAChRα7, seems to determine a cytokine reduction by the activation of microglia and astrocytes. Therefore, the decrease of such CNS inflammatory cytokines, interleukin (IL)1, IL6 and tumor necrosis factor (TNF), tends to increase neurogenesis and cell survival, preventing neuronal loss and minimizing neuroinflammation.

Nowadays, it is well-known that all components of the cholinergic system (i.e., ACh, nAChRs, muscarinic receptors, acetylcholinesterase - AChE) are present in most peripheral immune cells, including lymphocytes, macrophages, and dendritic cells, which contribute to the regulation of several immunological functions via the nAChRs [7–11]. Like microglia, also peripheral macrophages express nAChRα7, which, when activated, suppresses pro-inflammatory cytokine release [12].

Structurally, nAChRs are well-characterized membrane proteins, composed of five transmembrane subunits. Seventeen distinctive subunits were identified (α2–α10, β1–β4, γ, δ, and ε), which can be differently assembled to form either heteromeric (e.g., nAChRα4/β2) or homomeric pentamers (e.g., nAChRα7) [13].

As well as nAChRα7, nAChRα4/β2, which is a high-affinity binding protein, is also expressed in brain and peripheral immune cells and it is considered to have a major role in cognitive functions [14]. However, whether nAChRα4/β2 is involved in anti-inflammatory pathways remains poorly understood. Several studies reported that the activation of nAChRα4/β2 by the agonist nicotine suppressed IL1β and IL6, supporting the role of those receptors in neuroinflammation [15–17].

The increasing evidence that peripheral inflammation and neuroinflammation in the CNS are closely related suggests that altered peripheral inflammatory markers may unveil an underpinning neurodegenerative process [18]. Considering their accessibility and practicality, inflammatory markers in peripheral blood should be considered to monitor the presence of neuroinflammation in patients suffering from a neurodegenerative condition.

In the two most common neurodegenerative dementia i.e. Alzheimer’s Disease (AD) and Dementia with Lewy bodies (DLB), cortical cholinergic neurotransmission is progressively impaired, leading to the onset of cognitive and neuropsychiatric symptoms [19–22]. Therefore, the identification in human peripheral blood mononuclear cells (PBMCs) of different receptors (nAChR subtypes and all five muscarinic receptors) [23–25] may be correlated with their expression in brain regions [26]. Derangement of muscarinic leukocyte receptors has been previously observed in AD and DLB patients [27]. The present study aimes to characterize the expression level of nAChR subunits and of inflammatory cytokines in PBMCs of patients suffering from DLB and AD in comparison with healthy control (HC) subjects.

## Methods

### Patient recruitment and eligibility

Twenty-one patients diagnosed with probable DLB according to clinical criteria [28] (taking into account the one-year rule to rule out a diagnosis of Parkinson’s Disease with dementia) and thirteen patients with AD [29], frequency matched for gender, age, education, disease duration, and cognitive level, naïve to AChE inhibitors (AChEIs) treatment, were consecutively recruited from the Dementia Center of the Neurology Unit, “G. d’Annunzio” University of Chieti-Pescara. Eight HC, frequency matched for age and gender, were recruited among the patients’ spouses. Clinical assessment, including anthropometric measurements and physical examination, was performed at the baseline visit. In all patients enrolled, cognitive and neuropsychiatric profiles were also evaluated. The presence of fluctuating cognition (CF) was assessed by the CAF questionnaire [30], the presence of REM sleep behavior disorder (RBD) by the Mayo Questionnaire [31], parkinsonism was assessed by the Unified Parkinson’s Disease Rating Scale (UPDRS) score (part III) [32], and the presence of visual hallucinations (VH) by the Neuropsychiatric Inventory (NPI) [33]. Cognitive impairment was evaluated by Montreal Cognitive Assessment (MoCA) test [34]. For each participant with cognitive decline, the Clinical dementia rating (CDR) scale was also calculated [35].

This study has been conducted according to the Declaration of Helsinki and subsequent revisions and approved by the Ethics Committee at the University “G. d’Annunzio” Chieti-Pescara (Protocol code 2098 11/6/2020. Protocol “Neurodem” 26/7/2018, amend. 2/8/2018). All the participants or their caregivers signed informed consent to participate to the study.

### Sample collection

A blood sample was taken from each participant for biochemical and hematological measurements. Peripheral venous blood samples (10 mL) were collected in vacutainer tubes containing EDTA, according to the routine puncture method. Blood was layered over 5 mL of Ficoll-Paque (GE Healthcare, Merk, Darmstadt, Germany) and centrifuged at 1600 rpm for 40 min at room temperature. PBMCs were harvested from the interface, washed with Phosphate buffered saline (PBS, Merk, Darmstadt, Germany) and the cell pellet was resuspended in TRIzol reagent (Invitrogen, Life Technologies, Paisley, UK) and stored at − 80 °C for later analysis of gene expression.

### RNA extraction, RT and real-time PCR

Total RNA was extracted from PBMCs using QIAzol reagent (Qiagen, Hilden, Germany) according to the manufacturer’s protocol. The RNA concentration was determined by measuring the samples’ absorbance at 260 nm by NanoDrop 2000 UV-Vis Spectrophotometer (Thermo Scientific, Waltham, MA, USA) and its purity was assessed by the absorbance ratio 260/280 nm and 260/230 nm. For each sample, 1 μg of RNA was reverse transcribed into complementary DNA (cDNA) using QuantiTect Reverse Transcription Kit (Qiagen, Hilden, Germany). Subsequently, Real-Time PCR was performed using the GoTaq® qPCR Master Mix (Promega, Milan, Italy), to evaluate the gene expression (Table 1). All qRT-PCR reactions were performed in triplicates using the Mastercycler ep (Eppendorf, Hamburg, Germany) with the following conditions: initially, 2 min incubation at 95 °C followed by 40 cycles consisting in 30s 95°C, then 60 °C for 1 min and 30s at 68 °C. The gene expression analysis was done according to the ΔΔCt method [36].

**Table 1:** Gene sequence

Gene	Forward primer sequence (5′-3′)	Reverse primer sequence (5′-3′)
RPS18	CTTTGCCATCACTGCCATTAAG	TCCATCCTTTACATCCTTCTGTC
IL6	GTACATCCTCGACGGCATC	ACCTCAAACTCCAAAAGACCAG
TNFα	CCTTCCTGATCGTGGCAG	GCTTGAGGGTTTGCTACAAC
IL1β	TGAGGATGACTTGTTCTTTGAAG	GTGGTGGTCGGAGATTCG
nAChRα7	CTGCTCGTGGCTGAGATCAT	CTGGTCCACTTGGGCATCTT
nAChRα3	TCTGACTATGGTGGGGCAGA	CGTAGGACCAGGAACCGAAC
nAChRα4	TACTGTGTTCCCCGAGACGA	GCCACGTACTTCCAGTCCTC
nAChRβ2	TGGGTGAAGGTCGTCTTCC	CGACGTACTTCCAGTCCTCAC
nAChRβ4	GACCTATGACCACACGGAGATA	GAGATGAGCAGCAGGAAGAATG
AChE	GCGACTGATGCGATACTGG	CAGGTCCAGACTAACGTACTG

### Statistical analyses

Descriptive analysis was carried out using median and interquartile range (IQR) for the quantitative variables and percentages values for the qualitative ones. Despite transformation of quantitative data (logarithmic and boxcox), the Shapiro-Wilk test indicated that data were not normally distributed (*p* ≤ 0.05). For this reason, it was decided to leave the data in their original scale and to use non-parametric techniques.

The non-parametric Kruskal-Wallis test was performed to evaluate the differences between continuous variables and groups (DLB, AD and HC); Pearson’s chi-square test or Fisher’s exact test to evaluate the association between categorical variables and groups. The Dunn test, with Bonferroni’s correction, was calculated for the comparison between the pairs of medians for the identification of significant differences.

Non-parametric two sample Wilcoxon rank-sum (Mann-Whitney) test was used to compare cytokine gene expression between AD and DLB. Sign test was applied to evaluate the differences between AD and DLB levels vs HC.

Correlations among variables were tested using Spearman’s rho coefficients. For these analyses considering the explanatory nature of the study and the null hypothesis tested, we did not perform multiplicity adjustment.

Statistical significance was set at the level of ≤0.05, unless Bonferroni’s adjustment for multiple comparisons was needed (in this case the significance threshold was 0.0167 (p/k, assuming k = 3 contrast)). The power analyses assuming a large effect size showed adequate power (G∗Power 3.1.9.4). The analyses were performed using Stata software v17.1 (StataCorp, College Station, USA).

## Results

### Demographic and clinical characteristics

Table 2 reports full details of subject demographic and clinical characteristics. No significant differences were found for age, gender, and disease duration among groups. As expected, MoCA median scores were lower in DLB and AD patients compared to HC (*p* < 0.001).

**Table 2:** Demographic and clinical characteristics

	SW test; ***p***-value	DLB (***n*** = 21)	AD (***n*** = 13)	HC (***n*** = 8)	***p***-value
Age in years	W = 0.89; *p* = 0.001	81 (76–84)	79 (64–80)	74.5 (69.5–77.5)	0.060
Females	–	14 (66.6)	7 (53.8)	6 (75.0)	0.586
Education in years	W = 0.69; *p* = 0.003	8 (5–13)	5 (5–13)	9.5 (6.5–12)	0.464
Disease duration in years	W = 0.90; *p* = 0.007	4 (3–6.5)	6 (2.5–7.5)	–	0.456
MoCA score	W = 0.91; *p* = 0.008	11.0 (9.0–19.0)**	12.0 (8.0–15.0)**	29.0 (27.0–30.0)	< 0.001
CDR	–				
*1*		14 (66.7)	8 (61.5)	–	0.761
*2*		7 (33.3)	5 (38.5)	–	

N (%) or median and interquartile range (IQR) are shown when appropriate; *SW* Shapiro Wilk, *W* value test and *p*-value; *p*-value for Kruskal-Wallis’s test; **p-value <α/3 for Bonferroni multiple testing correction DLB and AD vs HC; *MoCA* Montreal Cognitive Assessment, *CDR* Clinical Dementia Rating

Clinical parameters for DLB patients showed that UPDRS-III median value was 23 (IQR 14–37); 19 patients presented parkinsonism (90.48%); 13 showed CF (61.9%); 16 presented VH (76.19%); 18 had RBD (85.71%).

### Inflammatory cytokine expression

**Fig. 1:**
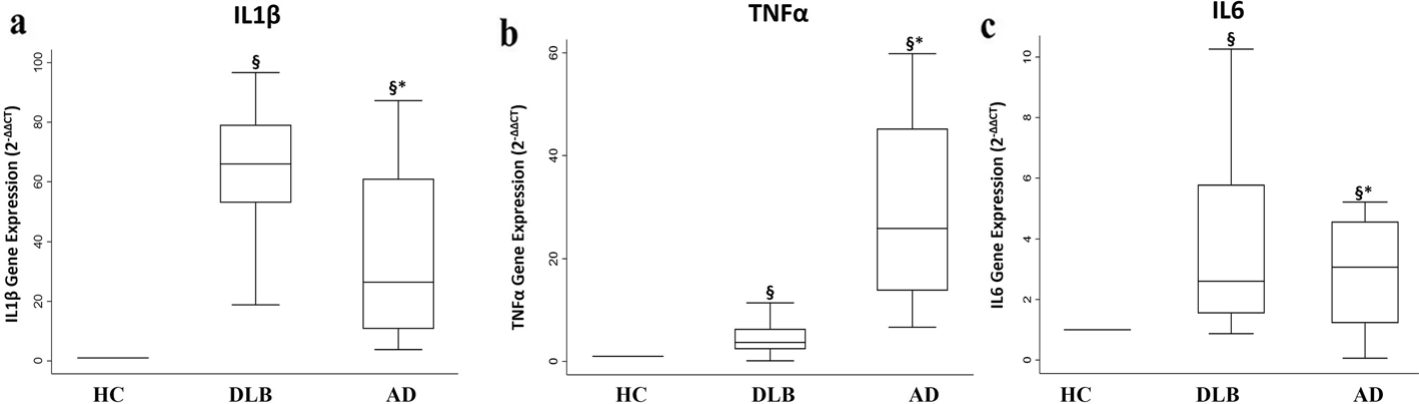
Inflammatory cytokines gene expression. Box plots show fold change (2^−ΔΔCt^) in expression through qRT-PCR in DLB and AD patients, both as relative to the group of HC. *P*-values determine the level of statistical significance in gene expression between the analyzed groups. **a** IL1β gene expression, SW test value = 0.80; *p* < 0.0001; *p-value derived from Mann-Whitney U test AD vs DLB *p* = 0.008; §*p*-value derived from Sign test DLB vs HC *p* < 0.0001; §*p*-value derived from Sign test AD vs HC *p* < 0.000.1 **b** TNFα gene expression, SW test value = 0.82; *p* = 0.001. *p-value derived from Mann-Whitney U test AD vs DLB *p* < 0.0001; §*p*-value derived from Sign test DLB vs HC *p* < 0.0001; §*p*-value derived from Sign test AD vs HC *p* < 0.0001. **c** IL6 gene expression, SW test value = 0.89; *p* = 0.005. *p-value derived from Mann-Whitney U test AD vs DLB *p* = 0.785; §*p*-value derived from Sign test DLB vs HC *p* < 0.0001; §*p*-value derived from Sign test AD vs HC *p* = 0.038

Overall, DLB and AD patients had higher IL1β gene expression than HC group. In detail, in DLB vs HC median was equal to 66.0 (IQR 52.9–79.1) and AD vs HC median was 26.4 (IQR 10.7–61.0). Moreover, IL1β gene levels were significantly higher in DLB than in AD (*p* = 0.008) (Fig. 1a). A higher gene expression of TNFα was also observed in DLB and AD patients compared to HC (DLB vs HC median = 3.7 (IQR 2.4–6.4); AD vs HC median = 25.9 (IQR 13.8–45.3). The expression of TNFα was significantly lower in DLB than in AD (*p* < 0.0001) (Fig. 1b).

Both DLB and AD groups showed higher IL6 gene expression with respect to control group (DLB vs HC median = 2.6 (IQR 1.5–5.8); AD vs HC median = 3.1 (IQR 1.2–4.6)). However, any significant difference was not detected between the two groups (*p* = 0.785) (Fig. 1c).

### nAChR subunits gene expression

**Fig. 2:**
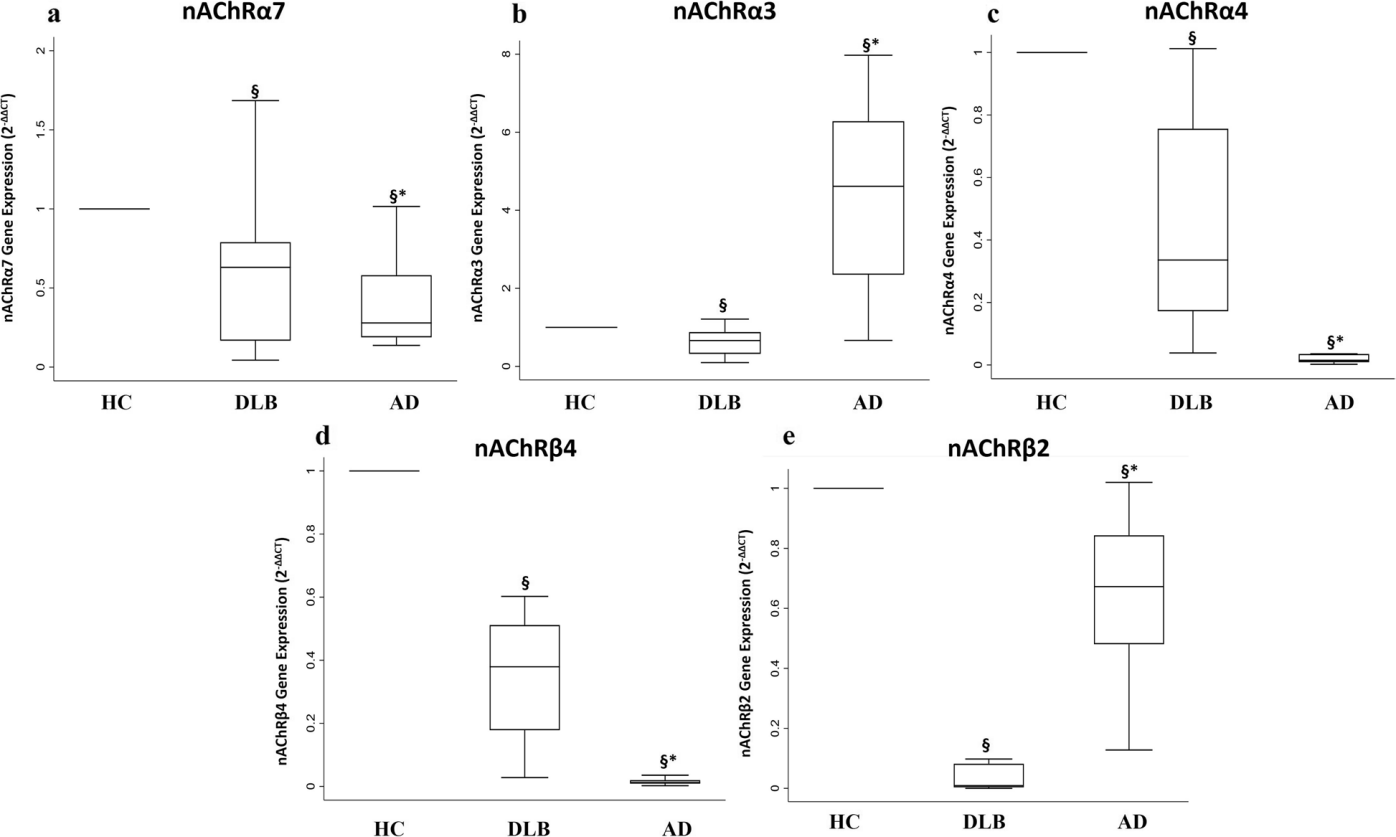
Nicotinic receptor subunit gene expression*.* Box plots show fold change (2^−ΔΔCt^) expression through qRT-PCR in DLB and AD patients, respect to the group of HC. **a** nAChRα7 gene expression, SW test value = 0.86, *p* = 0.007; *p-value derived from Mann-Whitney U test AD vs DLB *p* = 0.454; §*p*-value derived from Sign test DLB vs HC *p* = 0.002; §*p*-value derived from Sign test AD vs HC *p* = 0.021. **b** nAChRα3 gene expression, SW test value = 0.72, *p* < 0.0001; *p-value derived from Mann-Whitney U test AD vs DLB *p* < 0.001; §*p*-value derived from Sign test DLB vs HC *p* = 0.030; §*p*-value derived from Sign test AD vs HC *p* = 0.039. **c** nAChRα4 gene expression, SW test value = 0.71, *p* < 0.0001; *p-value derived from Mann-Whitney U test AD vs DLB *p* < 0.001; §*p*-value derived from Sign test DLB vs HC *p* = 0.011; §*p*-value derived from Sign test AD vs HC *p* = 0.002. **d** nAChRβ4 gene expression, SW test value = 0.66, *p* < 0.0001; *p-value derived from Mann-Whitney U test AD vs DLB *p* < 0.001; §*p*-value derived from Sign test DLB vs HC *p* = 0.012; §*p*-value derived from Sign test AD vs HC *p* = 0.001. **e** nAChRβ2 gene expression, SW test value = 0.90 *p* = 0.014. *p-value derived from Mann-Whitney U test AD vs DLB *p* < 0.001; §*p*-value derived from Sign test DLB vs HC *p* < 0.001; §*p*-value derived from Sign test AD vs HC *p* = 0.011

No significant differences were detected between HC and either DLB or AD in nAChRα7 gene expression (DLB vs HC (median = 0.6 (IQR 0.2–0.8); AD vs HC (median = 0.3 (IQR 0.2–0.6); AD vs DLB (*p* = 0.454)) (Fig. 2a). In DLB patients, the gene expression level of nAChRα3 was lower than in HC (median = 0.7 (IQR 0.3–0.9)) and in AD (median = 4.6 (IQR 2.3–6.3)) (*p* < 0.001) (Fig. 2b). Considering nAChRα4 gene expression, a decrease was observed in both DLB and AD patients compared to HC (DLB vs HC: median 0.3 (IQR 0.2–0.8)**;** AD vs HC: median = 0.0 (IQR 0.0–0.0)), although a significantly lower expression was detected in AD compared to DLB (*p* = 0.001) (Fig. 2c).

The nAChRβ4 gene expression was downregulated in both DLB and AD patients (DLB vs HC: median = 0.4 (IQR 0.2–0.5); AD vs HC: median = 0.0 (IQR 0.0–0.0)), and it was significantly lower in AD in comparison with DLB (*p* < 0.001) (Fig. 2d).

Respect to HC, nAChRβ2 gene expression was reduced in both DLB and AD patients (DLB vs HC: median = 0.0 (IQR 0.0–0.1); AD vs HC: median = 0.7 (IQR 0.5–0.8)), and significantly lower expression levels were observed in DLB compared to AD (*p* < 0.001) (Fig. 2e).

### AChE gene expression

**Fig. 3:**
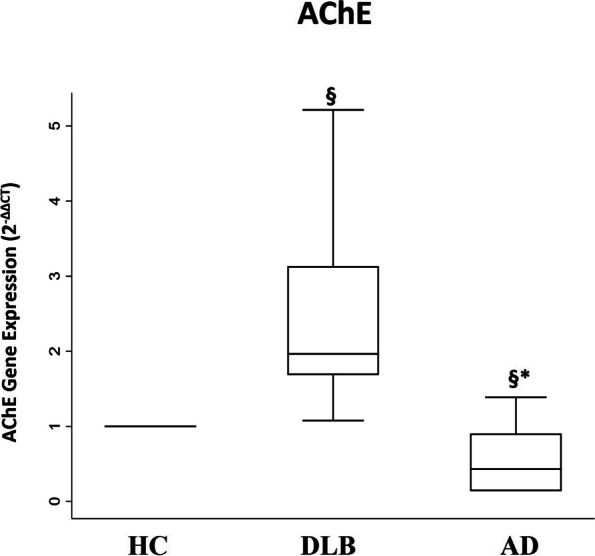
AChE gene expression, SW test value =0.85, *p* = 0.009; Box plots show fold change (2^−ΔΔCt^) expression through RT-qPCR in DLB and AD patients, respect to the group of HC. *p-value derived from Mann-Whitney U test AD vs DLB *p* = 0.002; §*p*-value derived from Sign test DLB vs HC *p* < 0.001; §*p*-value derived from Sign test AD vs HC *p* = 0.375

A different gene expression of AChE was detected in DLB vs AD patients (DLB median = 2.0 (IQR 1.7–3.1) vs AD median = 0.4 (IQR 0.1–0.9), *p* = 0.002) (Fig. 3).

### Correlation analysis

**Fig. 4:**
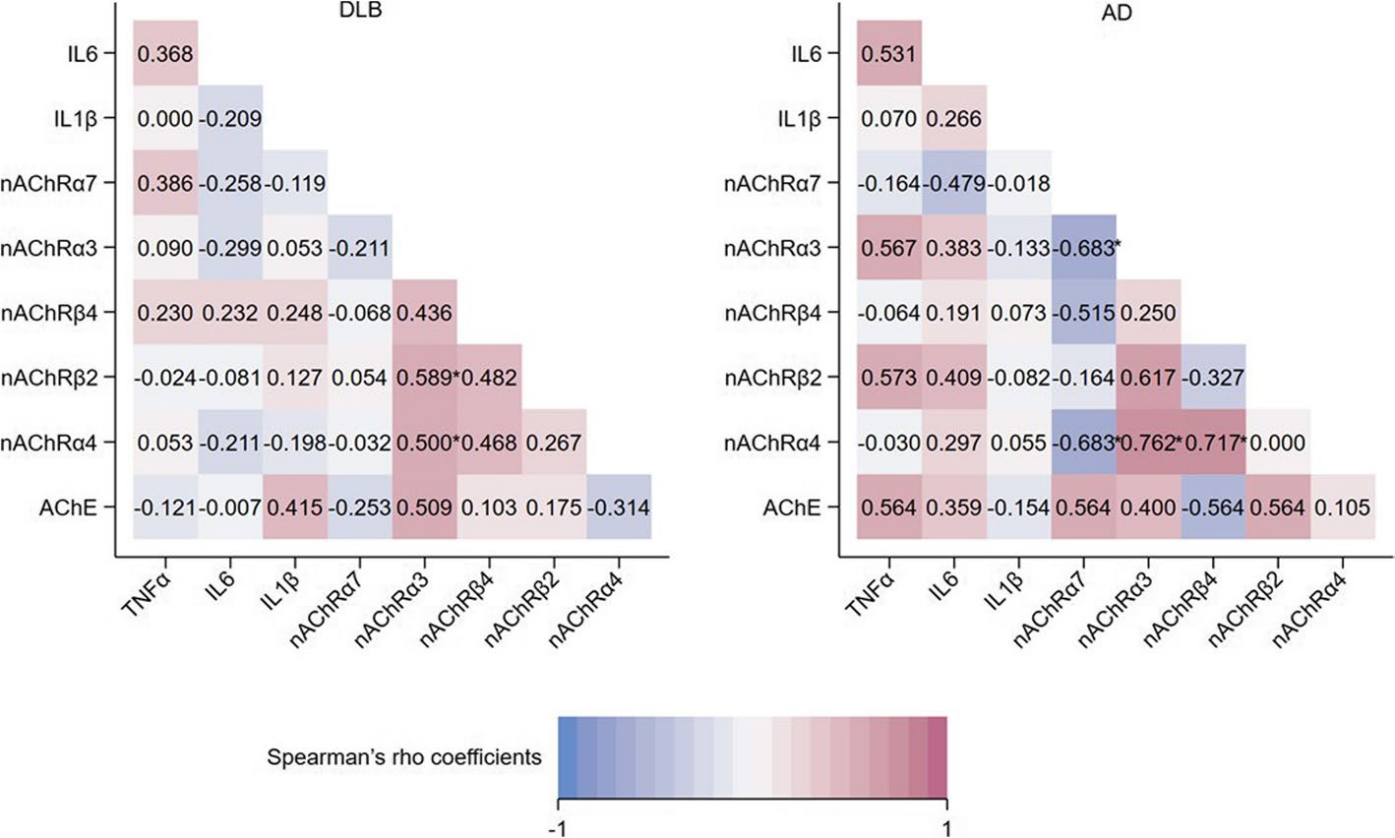
Matrix heatmaps represent correlations with Spearman’s rho between gene expression in DLB and AD groups (*p-value ≤0.05). The gradients in the heatmap show the strength of the correlation

Among several nAChR subtypes, a significant correlation was observed in both DLB and AD groups (Fig. 4). Indeed, in DLB group, a positive moderate correlation was detected between nAChRβ2 and nAChRα3 (rho = 0.589, *p* = 0.020), and between nAChRα4 and nAChRα3 (rho = 0.500, *p* = 0.041). In AD subjects, a positive correlation was also shown between nAChRα4 and nAChRα3 (rho = 0.762, *p* = 0.028) and between nAChRα4 and nAChRβ4 (rho = 0.717, *p* = 0.029), whereas a negative moderate correlation was observed between nAChRα7 and nAChRα3 (rho = − 0.683, *p* = 0.042) and between nAChRα7 and nAChRα4 (rho = − 0.683, *p* = 0.042).

## Discussion

Our results confirmed different peripheral inflammatory responses, as well as cholinergic involvement, in DLB and AD patients in comparison with HC subjects.

Pro-inflammatory cytokines were remarkably overexpressed in the two neurodegenerative dementias as compared to controls. Specifically, IL6, TNFα and IL1β gene expression was up regulated in both DLB and AD patients vs. HC. TNFα was however down-regulated in DLB vs.AD and IL1β was up-regulated in DLB compared to AD. These findings support the hypothesis that immune system might play a pivotal role in neurodegenerative pathways. Indeed, previous data reported peripheral inflammatory dysfunction in patients with mild cognitive impairment (MCI) or dementia [37, 38].

An increased microglia activation and, therefore, a pronounced production of cytokines have been observed in the brains of patients affected by DLB and AD [39, 40]. Some evidence suggested as pro-inflammatory cytokines secreted by microglial cells, including IL-1β, IL-6, and TNFα, may promote the overproduction of pathogenic β-amyloid proteins in AD brains [40]. An increased level of these pro-inflammatory cytokines was also observed in different DLB brain regions, suggesting a possible role in the spreading of alpha-synuclein aggregation in the CNS [39].

Pro-inflammatory cytokines seem to induce various intracellular signal transduction and metabolic pathways that, in combination with the expression of different nAChR subtypes, could produce distinctive immunological responses in DLB and AD individuals [27].

Indeed, differences in the glial response in AD vs. DLB have been suggested based on different cerebrospinal fluid (CSF) glial markers profiles [41].

In our study we did not find significant differences in the peripheral immunological patterns of cytokines between DLB and AD. This might be due to our small sample size or to the fact that our patients were at an overt stage of dementia, when differences in the pathophysiological mechanisms might be blurred by the higher levels of co-pathology.

However, an equally increased peripheral inflammation was recently reported in DLB and AD patients at the stage of mild cognitive impairment [38].

In the pathophysiology of cognitive decline, cholinergic impairment plays a central role in both AD and DLB conditions as a regulator of both neurodegenerative pathway and inflammatory response. Therefore, it is necessary clarify the interaction between cytokines and nAChRs [42, 43]. We measured the expression levels of nAChR subtypes, demonstrating different mRNA expressions among AD, DLB, and HC subjects. For all the nAChRs analyzed (i.e., nAChRα4, nAChRβ2 and nAChRβ4), the expression levels were lower in AD and DLB than HC subjects, whereas the gene expression of nAChRα3 was higher in AD and lower in DLB, respect to HC. No differences were found for nAChRα7 among groups.

The peripheral downregulation of nAChRs, observed in our cohort, may be related to the central cholinergic deficiency, which typically contribute to the pathogenesis of two diseases in study [42] but such an association needs to be deeply investigated as highlighted in the limitations of the study section.

Relatively greater losses of AChRs in DLB compared to AD have been reported in temporal and parietal neocortex and thalamic nuclei [44].

These characteristics are at the basis of the reported good symptomatic effect of AChEIs in DLB [45].

Additionally, our findings supported the hypothesis of an anti-inflammatory role for the cholinergic system; indeed, it was previously described as the binding of ACh to nAChRs may modulate the activity of immune cells, inhibiting cytokine synthesis and release [46, 47]. Our results support the effective interaction between cholinergic system and systemic inflammatory response since a peripheral derangement of nAChR expression correlated to increased levels of peripheral pro-inflammatory cytokines.

Therefore, in AD and DLB patients, the decrease of peripheral nAChR levels, as well as the activity of pro-inflammatory cytokines, might be considered as additional diagnostic biomarkers. The finding needs to be further investigated and validated in terms of diagnostic sensitivity and specificity in larger cohorts.

Notably, our results did not reveal a significant association among peripheral cytokines, nAChRs and clinical features (i.e., MoCA, CDR or disease duration), suggesting as this systemic proinflammatory activity may be considered as a marker of disease more than marker of disease stage or progression.

### Limitations of the study

Although the study reached its aim, there were some limitations.

The main constraint is related to the small cohort sample, which does not allow for generalizable considerations and to assess diagnostic sensitivity and specificity of the studied peripheral inflammatory markers. In addition, our results suggested a possible correlation between peripheral and central inflammation: but, further investigations should be focused on the assessment of the central immune system markers, measuring CSF cells, or performing imaging studies with microglia specific radiotracers, to better enforce the hypothesis of a direct correlation between neurodegeneration and both peripheral and central inflammation.

## Conclusions

In summary, the present study demonstrates an altered gene expression of pro-inflammatory cytokines and nAChR subunits from PBMCs of patients suffering from DLB and AD. These changes may reflect the CNS inflammatory activity involved in the neurodegenerative process. The measurement of these peripheral markers might be an attractive option to monitor cholinergic system dysfunction which may support clinical diagnoses and open the way for innovative treatment strategy.

Further investigations are necessary to understand if pro-inflammatory cytokines in DLB and AD individuals may differently modify receptor activity through a post-translational process that influence the distribution through the central and peripheral cholinergic system of nAChRs and their relative functions.

## Data Availability

The data supporting the results of this article are included within the article and can be required to the corresponding authors.

## Abbreviations

ACh: Acetylcholine
AChEIs: Acetylcholinesterase Inhibitors
AChE: Acetylcholinesterase
AChRs: ACh receptors
AD: Alzheimer’s disease
BBB: Blood-brain barrier
CNS: Central Nervous System
cDNA: Complementary DNA
DLB: Dementia with Lewy bodies
CF: Fluctuating cognition
HC: Healthy control
IL: Interleukin
IQR: Interquartile range
MoCA: Montreal Cognitive Assessment
NPI: Neuropsychiatric Inventory
nAChRs: Nicotinic cholinergic receptors
PBMCs: Peripheral blood mononuclear cells
PNS: Peripheral nervous system
RBD: REM sleep behavior disorder
SW: Shapiro Wilk
TNF: Tumor necrosis factor
UPDRS: Unified Parkinson’s Disease Rating Scale
VH: Visual hallucinations
